# Lemierre’s Syndrome Associated With Papillary Thyroid Cancer and Cerebellar Stroke

**DOI:** 10.7759/cureus.38889

**Published:** 2023-05-11

**Authors:** Dan C. S. Im, Vishaal Sridhar, Charles W Lanks

**Affiliations:** 1 Emergency Medicine, Harbor University of California Los Angeles Medical Center, Los Angeles, USA; 2 Pulmonary and Critical Care Medicine, Harbor University of California Los Angeles Medical Center, Los Angeles, USA; 3 Pulmonary and Critical Care Medicine, Lundquist Institute for Biomedical Innovation, Torrance, USA

**Keywords:** meningitis, fusobacteria, fusobacterium necrophorum, septic thrombophlebitis, jugular vein thrombosis, thyroid cancer, stroke, lemierre's syndrome

## Abstract

A 53-year-old woman with no past medical history presented to the Emergency Department with right frontal headache and ipsilateral neck pain. She was found to have right internal jugular vein thrombosis, right cerebellar stroke, meningitis, septic pulmonary emboli, and fusobacterium bacteremia, all consistent with a severe presentation of Lemierre’s syndrome (LS). While LS is often preceded by nasopharyngeal infection, no such history was elicited from our patient. Instead, concomitant papillary thyroid cancer with extension to her right internal jugular vein was implicated. Prompt recognition of these multiple related processes led to a timely initiation of appropriate therapy for infection, stroke, and malignancy.

## Introduction

Septic thrombophlebitis of the internal jugular vein, commonly referred to as Lemierre’s syndrome (LS) after Dr. André-Alfred Lemierre’s initial description in 1936, is typically preceded by nasopharyngeal infection, but it has been associated with many non-infectious pathologies as well. Here we present a rare case of LS associated with papillary thyroid cancer. Even more unusual, this patient developed ipsilateral cerebellar infarction as a result of retrograde venous obstruction. Although LS is characterized predominantly by local symptoms involving the neck, distant organ involvement is common as a result of both embolic spread via the venous circulation and hematogenous seeding as a result of bacteremia. Early recognition and prompt initiation of antibiotic therapy are the cornerstones of management in this disease.

## Case presentation

A 53-year-old woman with no known past medical history presented to the Emergency Department with a chief complaint of right frontal headache for three days. It was gradual in onset and progressively worsened over the previous three days. Her headache was associated with nausea, vomiting, and dizziness, and was exacerbated by movement of her head in any direction. She was evaluated by her primary care physician prior to presenting to the Emergency Department and reported being diagnosed with migraine headaches, although she was told at the time that she also had a fever.

At the time of presentation, she was afebrile, other vital signs were all within normal limits, and her physical examination revealed nuchal rigidity with active and passive flexion as well as severe tenderness to palpation over her right neck. An enlarged, but non-tender thyroid was palpable at the anterior neck with multiple enlarged non-tender cervical chain lymph nodes bilaterally. A comprehensive neurologic examination was performed. Strength and sensation were both 5/5 in all extremities, and her cranial nerve examination was unremarkable. She had right-sided dysmetria and gait instability. The remainder of her physical examination was unremarkable.

**Figure 1:**
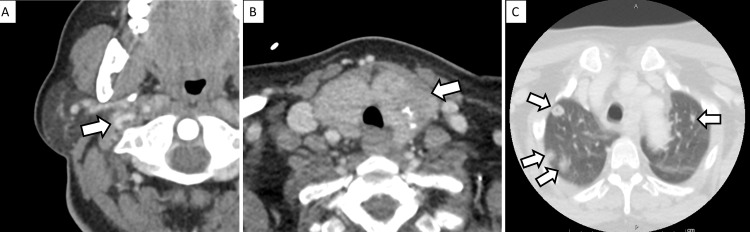
CT of the head and neck showing thrombosis of the right jugular vein, abnormal thyroid, and apical pulmonary nodules. CT of the head and neck with contrast demonstrating (A) thrombosis of the right internal jugular vein (white arrow), (B) an enlarged thyroid with scattered calcifications (white arrow), and (C) multiple apical pulmonary nodules, some with central cavitation (white arrows). CT, computed tomography

**Figure 2:**
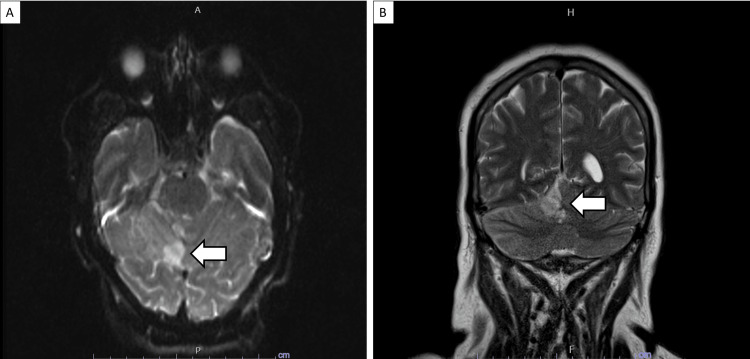
MRI of the brain showing right cerebellar stroke. MRI of the brain with and without gadolinium demonstrating right cerebellar infarction (white arrows) in the (A) axial DWI sequence and (B) coronal T2-weighted sequence. MRI, magnetic resonance imaging; DWI, diffusion-weighted imaging

A complete blood count was notable for a white blood cell count of 32,800 cells/mm^3^. Given the initial concern for bacterial meningitis, a lumbar puncture was performed, which revealed a cerebrospinal fluid (CSF) white blood cell count of 296 cells/mm^3^ (86% neutrophils, 11% lymphocytes), red blood cell count of 83 cells/mm^3^, protein of 131 mg/dL, glucose of 18 mg/dL, opening pressure of 33 cm H_2_O, and negative gram stain and culture. Computed tomography (CT) of the neck with contrast revealed thrombosis of the right internal jugular vein, multiple necrotic cervical lymph nodes, an enlarged thyroid gland with scattered calcifications, and several apical cavitary pulmonary nodules (Figure 1). Magnetic resonance imaging (MRI) of the brain with gadolinium revealed an acute right cerebellar stroke associated with non-occlusive right sigmoid sinus and internal jugular vein thrombosis (Figure 2). Shortly after the discovery of her acute cerebellar stroke, her mental status rapidly declined, with a repeat CT of her brain revealing increased edema with fourth ventricle obstruction and hydrocephalus. She was intubated for airway protection, and an external ventricular drain was placed. In light of neurosurgical intervention, anticoagulation for her stroke due to retrograde venous obstruction was not initiated.

Although initial blood cultures remained negative, metagenomic next-generation sequencing of microbial cell-free DNA (Karius Test™) in the blood was positive for both *Fusobacterium nucleatum* and *Porphyromonas gingivalis*, confirming the diagnosis of LS. An ultrasound-guided fine needle aspiration of a cervical chain lymph node revealed papillary thyroid carcinoma, stage pT3bN1aM0. She was treated with ceftriaxone and metronidazole, and with resolution of her elevated intracranial pressure, she made rapid improvement and was successfully extubated. She ultimately regained significant function but had persistent ataxia and difficulty with ambulation. She was discharged to an outpatient rehabilitation facility where she completed a six-week course of ceftriaxone and metronidazole. Her lung nodules were too small to biopsy, but they improved with antibiotics on follow-up chest imaging. A total thyroidectomy was subsequently performed, and her thyroid cancer was treated with radioactive iodine therapy.

## Discussion

Critically ill patients often present with several seemingly unrelated symptoms that coalesce into a single, unifying diagnosis. André-Alfred Lemierre described one such syndrome in 1936, characterized by nasopharyngeal infection, *Fusobacterium necrophorum* septicemia, and internal jugular vein thrombosis [1]. In addition to these, our patient also had septic emboli to her bilateral lungs, a commonly described finding in LS, as well as an acute right cerebellar stroke caused by retrograde venous obstruction, a highly unusual finding.

Fusobacteria species account for more than 50% of cases with *F. necrophorum,* representing the most common causative pathogen in LS [1]. Other causative organisms include Streptococcus spp, *Staphylococcus* spp, *Porphyromonas* spp, *Enterobacteriaceae*, and *Bacteroides*, among others [2]. Fusobacteria are anaerobic gram-negative bacilli commonly found in normal oral flora. In the past, fusobacteria have been difficult to identify in culture, but the increasing use of microbial cell-free DNA sequencing has the potential to significantly increase sensitivity, although data on sensitivity and clinical application are still lacking. In our patient, two causative organisms, *Fusobacterium nucleatum* and *Porphyromonas gingivalis*, were ultimately identified using this method, even after receiving antibiotics.

LS affects only one person per million per year, and patients are typically between 16 and 30 years of age [2]. Pathogenesis often begins with primary head and neck infection. While Lemierre’s initial case series exclusively described patients with nasopharyngeal infections, it is now recognized that infections of adjacent structures including sinusitis, otitis, and parotitis have similar potential to cause internal jugular vein thrombosis. In rare cases, no precipitating oropharyngeal infection is identified, and other head and neck processes including trauma, malignancy, and vasculitis may be implicated [3,4]. Following disruption of local mucosal barriers, the in vitro platelet aggregation and bacterial toxin production have been implicated as the pathogenesis, although the exact mechanism is unknown [5].

Most patients have presenting complaints related to the site of their primary infection, but some initially present with complaints related to thrombophlebitis of the internal jugular vein such as neck pain and swelling. Emboli from the thrombosed jugular vein are typically confined to the venous circulation, and the lungs are the most commonly affected organ system [2]. Hematogenous seeding may also occur, affecting distant sites such as the joints, liver, kidneys, spleen, soft tissue, and bones. In this case, non-tender bilateral lymphadenopathy coupled with abnormal imaging of the thyroid gland was uncharacteristic and prompted further investigation. Central nervous system involvement is exceedingly rare and only described in a small number of case reports [6]. Associated meningitis, which our patient had, is related to increased risk for thrombus extension to the cerebral veins [7]. Malignancy, particularly head and neck cancer, is also a known risk factor for thrombosis of cerebral veins and stroke in the setting of hypercoagulability [8].

The diagnosis of LS relies on both the isolation of a causative organism and imaging of the neck with ultrasound, CT, or MRI [6]. Fusobacteria are difficult to culture, and the true incidence of culture-negative LS is unknown. Microbial cell-free DNA sequencing has already revolutionized pathogen detection in a number of infectious processes, and while the impact of this technology on the diagnosis of LS is unclear, the identification of fusobacteria in patients with otherwise sterile cultures has already been described in other diseases [9]. Imaging of the jugular vein with ultrasound is a cost-effective and non-invasive means of diagnosing venous thrombosis [6]. However, CT imaging has the added benefit of being able to assess adjacent structures, such as the lungs, potentially revealing the presence of distant venous emboli or sites of hematogenous spread. In our patient, imaging of the neck was performed initially, and apical pulmonary cavities were noted. CT imaging also revealed an abnormal thyroid gland with calcifications and bilateral necrotic lymphadenopathy highly suspicious for malignancy.

Antibiotics are the mainstay of treatment in LS, although an optimal regimen has not been adequately studied in randomized trials. Metronidazole is highly active against all fusobacterium species but is rarely used as monotherapy. Because of the existence of beta-lactamase producing strains of *F. necrophorum*, a beta-lactamase resistant regimen should be chosen [10]. Empiric regimens include piperacillin-tazobactam, carbapenems, or ceftriaxone in combination with metronidazole. While the duration of therapy is uncertain, it typically depends on the severity of disease and the presence of complications such as distant emboli or hematogenous spread. It should be noted that because causative organisms were identified by metagenomic testing only, antibiotic susceptibility data were not available. Our patient was managed with a six-week course of ceftriaxone and metronidazole, which led to improvement in symptoms and radiographic abnormalities.

The use of systemic anticoagulation in LS remains controversial, and there are no randomized trials addressing this issue. Although anticoagulation may be of some benefit in other types of septic thrombophlebitis, randomized data supporting these practices is also lacking. Anticoagulation is used more frequently in patients with retrograde extension of jugular vein thrombosis but has not been associated with improved outcomes in any patients with septic thrombophlebitis of the internal jugular vein [11]. In our patient, magnetic resonance venography was repeated after three months, which demonstrated interval resolution of intracranial thrombosis without anticoagulation.

## Conclusions

LS can rarely present as a complication of anatomic abnormalities but has not previously been described in association with undiagnosed thyroid malignancy. Although the central nervous system is rarely involved in LS, hypercoagulability of malignancy likely contributed to an acute ipsilateral cerebellar stroke due to retrograde venous obstruction.

## References

[1] Lemierre A. (1936 (1936). On certain septicaemia due to anaerobic organisms. Lancet.

[2] Johannesen KM, Bodtger U (2016). Lemierre's syndrome: current perspectives on diagnosis and management. Infect Drug Resist.

[3] Denesopolis JM, Medicherla Singh RC, Shah AR, Lyon R, Chao E, Hochsztein JG, Rivera A (2020). A unique case of Lemierre's syndrome status post blunt cervical trauma. Vascular.

[4] Osman M, Hasan S, Bachuwa G (2017). Oesophageal cancer presenting as Lemierre's syndrome caused by Streptococcus anginosus. BMJ Case Rep.

[5] Riordan T, Wilson M (2004). Lemierre's syndrome: more than a historical curiosa. Postgrad Med J.

[6] Kuppalli K, Livorsi D, Talati NJ, Osborn M (2012). Lemierre's syndrome due to Fusobacterium necrophorum. Lancet Infect Dis.

[7] Bader-Meunier B, Pinto G, Tardieu M, Pariente D, Bobin S, Dommergues JP (1994). Mastoiditis, meningitis and venous sinus thrombosis caused by Fusobacterium necrophorum. Eur J Pediatr.

[8] Duman T, Uluduz D, Midi I (2017). A multicenter study of 1144 patients with cerebral venous thrombosis: the VENOST study. J Stroke Cerebrovasc Dis.

[9] Zafar H, Rashid MU, Mandzhieva B, Shobar R, Jain AG (2020). Multiloculated liver abscess caused by fusobacterium: role of Karius testing in diagnosis. Cureus.

[10] Brook I (1993). Infections caused by beta-lactamase-producing Fusobacterium spp. in children. Pediatr Infect Dis J.

[11] Armstrong AW, Spooner K, Sanders JW (2000). Lemierre's syndrome. Curr Infect Dis Rep.

